# PDE4 inhibitor rolipram inhibits the expression of microsomal prostaglandin E synthase‐1 by a mechanism dependent on MAP kinase phosphatase‐1

**DOI:** 10.1002/prp2.363

**Published:** 2017-10-18

**Authors:** Lauri Tuure, Mari Hämäläinen, Eeva Moilanen

**Affiliations:** ^1^ The Immunopharmacology Research Group Faculty of Medicine and Life Sciences University of Tampere and Tampere University Hospital Tampere Finland

**Keywords:** MAP kinases, MKP‐1, mPGES‐1, PDE4

## Abstract

Phosphodiesterase‐4 (PDE4) inhibitors have recently been introduced to the treatment of COPD and psoriatic arthritis. Microsomal prostaglandin E synthase‐1 (mPGES‐1) is an inducible enzyme synthesizing PGE_2_, the most abundant prostanoid related to inflammation and inflammatory pain. mPGES‐1 is a potential drug target for novel anti‐inflammatory treatments aiming at an improved safety profile as compared to NSAIDs. Here we investigated the effect of the PDE4 inhibitor rolipram on the expression of mPGES‐1 in macrophages; and a potential mediator role in the process for MAP kinase phosphatase‐1 (MKP‐1) which is an endogenous factor limiting the activity of the proinflammatory MAP kinases p38 and JNK. The expression of mPGES‐1 was decreased, whereas that of MKP‐1 was enhanced by rolipram in wild‐type murine macrophages. Interestingly, rolipram did not reduce mPGES‐1 expression in peritoneal macrophages from MKP‐1‐deficient mice. A reduced phosphorylation of JNK, but not p38 MAP kinase, was specifically associated with the decreased expression of mPGES‐1. Accordingly, mPGES‐1 expression was suppressed by JNK but not p38 inhibitor. These findings underline the significance of the increased MKP‐1 expression and decreased JNK phosphorylation associated with the downregulated expression of mPGES‐1 by PDE4 inhibitors in inflammation.

**Abbreviations**
cAMP: cyclic AMP
COXs: cyclo‐oxygenases
DMSO: dimethyl sulfoxide
EDTA: ethylenediaminetetraacetic acid
FBS: fetal bovine serum
LPS: lipopolysaccharide
MAPK: mitogen‐activated protein kinase
MKP‐1: mitogen‐activated protein kinase phosphatase 1
mPGES‐1: microsomal prostaglandin E synthase‐1
PBS: phosphate‐buffered saline
PDE4: phosphodiesterase‐4
PGE_2_: prostaglandin E_2_

## Introduction

Phosphodiesterase‐4 (PDE4) is a family of enzymes expressed widely in immune cells, including macrophages. PDE4 enzymes catalyze the degradation of cyclic AMP (cAMP), modulate immune cell functions and have an essential role in inflammation (Gantner et al. 1997; Essayan 1999; Spina 2008). Specific inhibitors of PDE4 are novel anti‐inflammatory drugs, of which roflumilast was lately introduced for the treatment of COPD and apremilast for plaque psoriasis and psoriatic arthritis (Lipworth 2005; Rabe 2011; Kavanaugh et al. 2015; Papp et al. 2015). Recently, PDE4 was also presented as a beneficial drug target in B‐cell lymphoma, based on the findings of the anti‐inflammatory properties of PDE4 inhibitors (Cooney and Aguiar 2016).

Rolipram is a selective PDE4 inhibitor, the anti‐inflammatory effects of which have been shown to be, at least partly, dependent on the MAP kinase phosphatase‐1 (MKP‐1) (Lee et al. 2012; Korhonen et al. 2013; Patel et al. 2015). MKP‐1 is an endogenous enzyme able to dephosphorylate and hence inactivate the proinflammatory MAP kinase p38 and JNK pathways (Franklin and Kraft 1997; Chi et al. 2006; Hammer et al. 2006; Zhao et al. 2006; Korhonen et al. 2011). MKP‐1 expression is enhanced by various inflammatory factors and serves as a limiting factor against excessive inflammatory response (Korhonen and Moilanen 2014). In addition, some anti‐inflammatory compounds further enhance MKP‐1 expression or activity leading to substantial suppression of inflammatory responses (Kassel et al. 2001; Nieminen et al. 2010).

mPGES‐1 has evoked interests as a potential anti‐inflammatory drug target since it was characterized in 1999 (Jakobsson et al. 1999; Samuelsson et al. 2007). It is an inducible enzyme catalyzing the synthesis of prostaglandin E_2_ (PGE_2_), situated downstream of cyclo‐oxygenases (COXs) in the prostaglandin synthesis pathway. It has been therefore suggested that by inhibiting the activity or expression of mPGES‐1, it would be possible to achieve therapeutic effects comparable with COX‐inhibitors (i.e., nonsteroidal anti‐inflammatory drugs, NSAIDS) but with less adverse effects (Samuelsson et al. 2007; Korotkova and Jakobsson 2014; Koeberle and Werz 2015). Several inhibitors of the activity of mPGES‐1 have been characterized with a promising preclinical profile but unfortunately, none of them is available for clinical use so far (Koeberle and Werz 2015). Interestingly, the effects of mPGES‐1 inhibitors may not be limited to inhibition of PGE_2_ synthesis or classical effects of PGE_2_ in inflammation. For instance, Idborg et al. (2013) recently reported that genetic deletion of mPGES‐1 shifted eicosanoid profiles toward anti‐inflammatory direction in activated macrophages by reducing PGE_2_ production and enhancing PGD_2_ metabolites and some anti‐inflammatory fatty acids. Also, Raouf et al. (2016) reported that platelet activation associated with inflammation is tempered in mPGES‐1‐deficient mice as compared to wild‐type animals.

Another pharmacological approach could be inhibition of mPGES‐1 expression in inflammatory conditions. The regulation of mPGES‐1 is not known in detail but it has been shown to be suppressed by anti‐inflammatory drugs dexamethasone and aurothiomalate (Stichtenoth et al. 2001; Korotkova et al. 2005; Tuure et al. 2015). In addition, mPGES‐1 expression has been reported to be upregulated by p38 and/or JNK MAP kinases. (Han et al. 2002; Masuko‐Hongo et al. 2004; Degousee et al. 2006; de Oliveira et al. 2008; Båge et al. 2010; He et al. 2016). On the other hand, anti‐inflammatory effects of the PDE4 inhibitor rolipram (Korhonen et al. 2013), like those of dexamethasone (Kassel et al. 2001; Abraham et al. 2006; Shipp et al. 2010) and aurothiomalate (Nieminen et al. 2010) have been shown to be mediated by enhanced expression of the anti‐inflammatory phosphatase MKP‐1 which leads to reduced activity of MAP kinases through dephosphorylation. We therefore aimed to study if the PDE4 inhibitor rolipram downregulates the expression of mPGES‐1 and if MKP‐1 is involved in mediating the effect.

There are no published reports on the effects of PDE4 inhibitors on the expression of mPGES‐1 and neither those on the role of MKP‐1 in the expression of mPGES‐1. The knowledge about interactions of phosphorylated MAP kinases and the expression of mPGES‐1 is also limited. Therefore, we investigated the effects of the selective PDE4 inhibitor rolipram on the expression of mPGES‐1 and MKP‐1 and on the (de)phosphorylation of MAP kinases p38 and JNK in classically activated macrophages using the J774 murine macrophage cell line. Moreover, we tested the hypothesis that the effects of rolipram on the expression of mPGES‐1 could be mediated by MKP‐1 by studying the effects of rolipram on peritoneal macrophages from MKP‐1‐deficient and corresponding wild‐type mice.

## Materials and Methods

### Cell culture

J774 mouse macrophages (American Type Culture Collection, Rockville Pike, MD, USA) were cultured at +37°C in 5% CO_2_ atmosphere in Dulbecco's Modified Eagle's medium (DMEM; Invitrogen, Paisley, UK) containing 10% (v/v) heat‐inactivated fetal bovine serum (FBS), 100 U/mL penicillin, 100 *μ*g/mL streptomycin and 250 ng/mL amphotericin B (all from Gibco, Wien, Austria). 2 × 10^5^ cells per well were seeded on 24‐well plates and the cell monolayers were grown for 72 h to confluence prior to the experiments. SP600125 (Sigma‐Aldrich Inc., St. Louis, MO, USA) and BIRB796 (Axon MedChem, Groningen, Netherlands) were dissolved in dimethyl sulfoxide (DMSO) and rolipram (Axon MedChem), dexamethasone (Orion Corp., Espoo, Finland) and lipopolysaccharide (LPS) from *Escerichia coli* strain 0111:B4 (Sigma‐Aldrich Inc.) in phosphate‐buffered saline (PBS). LPS and the compounds of interest in concentrations indicated or the solvent were added to the cells in fresh culture medium containing 10% FBS and the supplements, and the final concentration of DMSO was adjusted to 0.1% in all wells. Cells were further incubated for the time indicated before collecting cellular proteins/RNA and the culture medium.

Mouse peritoneal macrophages were harvested from MKP‐1‐deficient and corresponding wild‐type C57BL/6 mice originally generated in the laboratory of R. Bravo at Bristol‐Myers Squibb Pharmaceutical Research Institute (Princeton, NJ, USA). Mice were bred at the University of Tampere animal facility under standard conditions (12:12 light:dark cycle, +22 ± 1°C temperature, 50–60% humidity), and food and water provided ad libitum. Animal experiments were carried out in accordance with the legislation for the protection of animals used for scientific purposes (Directive 2010/63/EU), and the study was approved by the National Animal Experiment Board.

Peritoneal macrophages were obtained by peritoneal lavage with sterile PBS supplemented with 0.2 mmol/L ethylenediaminetetraacetic acid (EDTA; Sigma‐Aldrich Inc.). Cells were washed and seeded on 24‐well plates (1 × 10^6^ cells/well) in RPMI medium supplemented with 2% FBS, 100 U/mL penicillin, 100 *μ*g/mL streptomycin and 250 ng/mL amphotericin B. Cells were incubated overnight, washed and treated with the compounds of interest for the time indicated.

### Preparation of cell lysates and Western blot analysis

At the indicated time‐points, the culture medium was removed from the cells. Cells were washed with ice‐cold PBS and solubilized in cold lysis buffer containing 10 mmol/L Tris–HCl, 5 mmol/L EDTA, 50 mmol/L NaCl, 1% Triton X‐100, 0.5 mmol/L phenylmethylsulfonyl fluoride, 1 mmol/L sodium orthovanadate, 20 *μ*g/mL leupeptin, 50 *μ*g/mL aprotinin, 5 mmol/L sodium fluoride, 2 mmol/L sodium pyrophosphate and 10 *μ*mol/L *n*‐octyl‐*β*‐D‐glucopyranoside (all from Sigma‐Aldrich Inc.). After incubation for 15 min on ice, lysates were centrifuged, and the supernatants were collected and mixed in a ratio of 1:4, with SDS loading buffer [62.5 mmol/L Tris–HCl, pH 6.8, 10% glycerol, 2% SDS, 0.025% bromophenol blue and 5% *β*‐mercaptoethanol (all from Sigma‐Aldrich Inc)], and stored at −20°C until analyzed. Equal amounts of protein (10 or 20 *μ*g) were loaded on a 12% SDS‐polyacrylamide gel and separated by electrophoresis. Proteins were transferred to nitrocellulose membranes by dry electroblotting using iBlot gel transfer stacks and the Invitrogen iBlot Device (Invitrogen, Carlsbad, CA, USA) according to the manufacturer's instructions. After transfer, the membrane was blocked in TBS/T [20 mmol/L Trisbase (pH 7.6), 150 mmol/L NaCl, 0.1% Tween‐20] containing 5% non‐fat milk for 1 h at room temperature. For detection of phosphorylated proteins, membranes were blocked in TBS/T containing 5% BSA. Membranes were incubated overnight at +4°C with primary antibody and for 1 h with secondary antibody, and the chemiluminescent signal was detected by ImageQuant™ LAS 4000 mini (GE Healthcare Bio‐Sciences AB, Uppsala, Sweden). The chemiluminescent signal was quantified with ImageQuant TL 7.0 Image Analysis Software (GE Healthcare Bio‐Sciences AB). Following antibodies were used in the Western blot analysis: mPGES‐1 antibody (AS‐03031; Agrisera AB, Vännäs, Sweden), actin antibody (sc‐1616R, Santa Cruz Biotechnology, CA, USA), JNK antibody (#9251, Cell Signaling Technology Inc., Beverly, MA, USA) and polyclonal goat anti‐rabbit antibody (sc‐2004; Santa Cruz Biotechnology), MKP‐1 antibody (SAB2500331; Sigma‐Aldrich Inc), p38 MAPK antibody (ab27986; Abcam plc., Cambridge, UK), phospho‐p38 MAPK antibody (#9211, Cell Signaling Technology Inc), and phospho‐JNK antibody (#9251; Cell Signaling Technology Inc).

### RNA extraction and quantitative reverse transcription polymerase chain reaction (qRT‐PCR)

At the indicated time‐points, the culture medium was removed, and cell homogenization and RNA extraction were carried out using GenElute™ Mammalian Total RNA Miniprep Kit (Sigma‐Aldrich Inc.) according to the manufacturer's instruction. Reverse transcription of RNA extracted from J774 cells and from peritoneal macrophages to cDNA was performed by TaqMan^®^ Reverse Transcription Reagents (Applied Biosystems, Foster City, CA, USA) and Maxima First strand cDNA synthesis kit for RT‐qPCR (Thermo Fisher Scientific, Waltham, MA, USA), respectively. Primers and probes were purchased from Metabion (Martinsried, Germany). Their sequences and concentrations were optimized according to the manufacturer's guidelines in TaqMan Universal PCR Master Mix Protocol part number 4304449 revision C (Applied Biosystems) and were as follows: mouse mPGES‐1 CCTGGATACATTTCCTCGTTGTC (forward, 300 nmol/L), GAAGGCGTGGGTTCAGCTT (reverse, 300 nmol/L), ACAGGCCGTGTGGTACACACCG (probe, 150 nmol/L); mouse MKP‐1 CTCCTGGTTCAACGAGGCTATT (forward, 300 nmol/L), TGCCGGCCTGGCAAT (reverse, 300 nmol/L), CCATCAAGGATGCTGGAGGGAGAGTGTT (probe, 150 nmol/L); and mouse GAPDH GCATGGCCTTCCGTGTTC (forward, 300 nmol/L), GATGTCATCATACTTGGCAGGTTT (reverse, 300 nmol/L) and TCGTGGATCTGACGTGCCGCC (probe, 150 nmol/L). Quantitative PCR was performed using TaqMan Universal PCR Master Mix and ABI Prism 7500 sequence detection system (Applied Biosystems). The PCR cycling parameters were incubation at 50°C for 2 min, incubation at 95°C for 10 min, 40 cycles of denaturation at 95°C for 15 sec and annealing and extension at 60°C for 1 min. A standard curve method was used to estimate the relative mRNA levels. When calculating the results, mPGES‐1 and MKP‐1 mRNA levels were first normalized against GAPDH.

### Statistics

Results are expressed as mean + standard error of the mean (SEM). One‐way ANOVA with Bonferroni's posttest was performed using GraphPad InStat version 3.10 for Windows. Differences were considered significant at *P <* *0.05, **P <* *0.01 and ***P <* *0.001.

## Results

**Figure 1:**
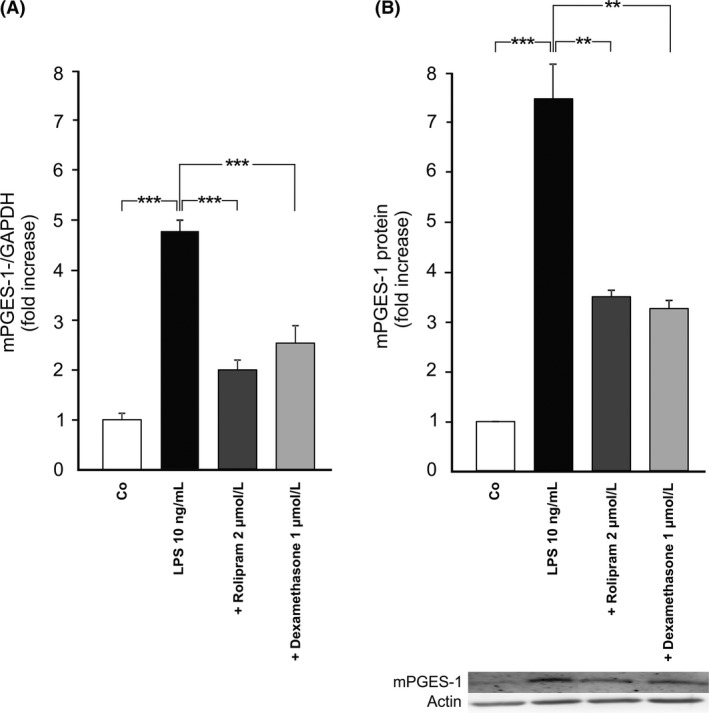
Effects of the PDE4 inhibitor rolipram and dexamethasone on mPGES‐1 expression in murine J774 macrophages. Cells were incubated with bacterial lipopolysaccharide (LPS) and rolipram or dexamethasone (which was used as a control compound) for 24 h. mPGES‐1 mRNA levels were measured by quantitative RT‐PCR and normalized against GAPDH mRNA levels (A). mPGES‐1 protein expression was measured by Western blot where actin was used as a loading control (B). mPGES‐1 expression levels in unstimulated (control) cells were set as 1 and the other values were related to that. Results are expressed as mean + SEM,* n* = 6–7 (A) and 5–6 (B). One‐way ANOVA with Bonferroni's post‐test was performed and statistical significance is indicated as **P < 0.01 and ***P < 0.001.

An evident increase in the expression of mPGES‐1 was found when J774 macrophages were stimulated with LPS (Fig. 1). The PDE4 inhibitor rolipram significantly inhibited the expression of mPGES‐1 mRNA and protein in stimulated cells, which is an original finding. The glucocorticoid dexamethasone was used as a positive control for inhibition of mPGES‐1 expression (Stichtenoth et al. 2001) and it also decreased the expression of mPGES‐1 as expected.

**Figure 2:**
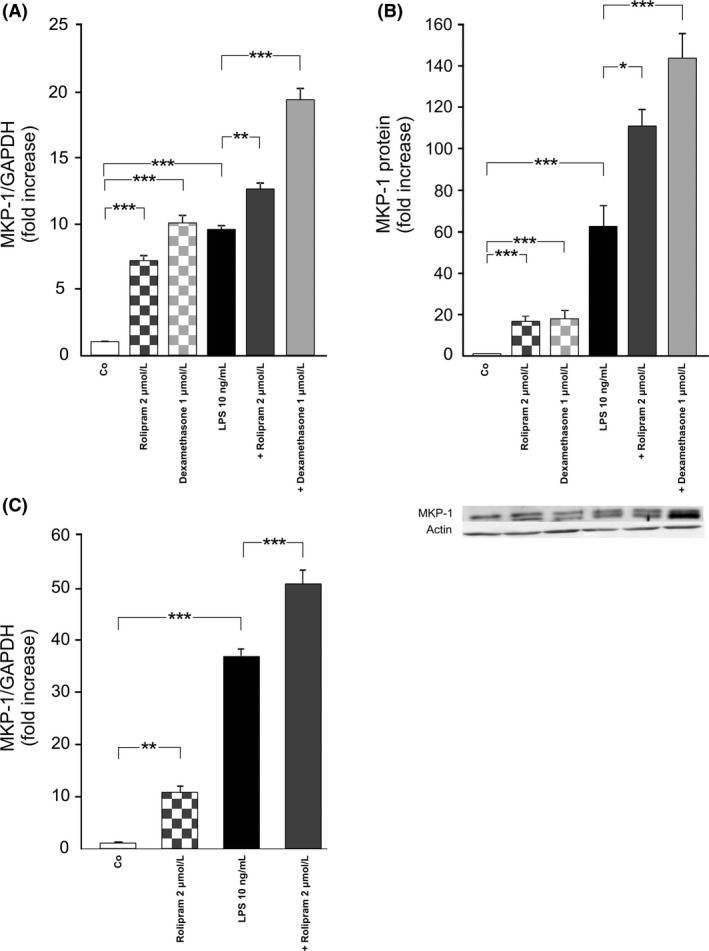
Effects of rolipram and dexamethasone on the production of MKP‐1 in murine J774 macrophages (A and B) and in mouse peritoneal macrophages (C). Cells were incubated with rolipram or dexamethasone (which was used as a control compound) with and without bacterial lipopolysaccharide (LPS) for 1 h. MKP‐1 mRNA levels were measured by quantitative RT‐PCR and normalized against GAPDH mRNA levels (A and C). MKP‐1 protein expression was measured by Western blot where actin was used as a loading control (B). MKP‐1 expression levels in the unstimulated (control) cells were set as 1 and the other values were related to that. Results are expressed as mean + SEM,* n* = 11–12 (A), 9 (B) and 5–6 (C). One‐way ANOVA with Bonferroni's post‐test was performed and statistical significance is indicated as *P < 0.05, **P < 0.01 and ***P < 0.001.

To test the hypothesis that the inhibition of the expression of mPGES‐1 by rolipram could be mediated by MKP‐1, we first measured the effects of rolipram on the expression of MKP‐1. Rolipram significantly increased the expression of MKP‐1 mRNA and protein both in the absence and in the presence of LPS as compared to the unstimulated cells (Fig. 2). Dexamethasone was used as a positive control (Kassel et al. 2001) and it also enhanced MKP‐1 levels as expected.

**Figure 3:**
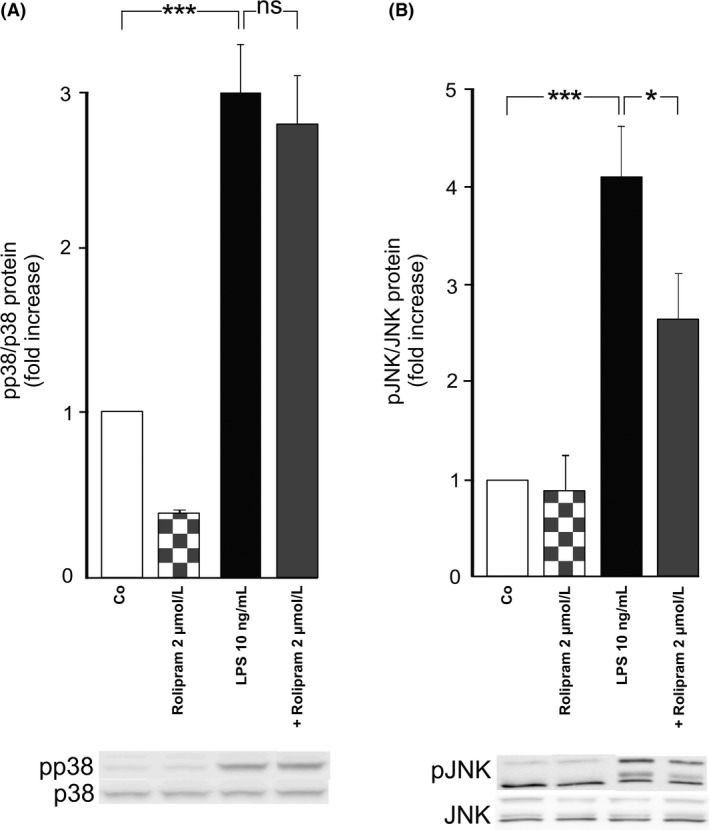
Effects of rolipram on the phosphorylation of MAP kinases p38 (A) and JNK (B). J774 cells were preincubated with the rolipram for 1 h and stimulated with bacterial lipopolysaccharide (LPS) for 30 min. The levels of phosphorylated and total p38 and JNK were measured by Western blot and the amounts of phosphorylated p38 and JNK were normalized against the total p38 and JNK, respectively. Levels of pp38 and pJNK in the unstimulated (control) cells were set as 1 and the other values were related to that. Results are expressed as mean + SEM,* n* = 4 (A) and 6 (B). One‐way ANOVA with Bonferroni's post‐test was performed and statistical significance is indicated as *P < 0.05, ***P < 0.001 and ns = not significant.

MKP‐1 is known to control the intensity of inflammation by dephosphorylating MAP kinase p38, JNK or both depending on the cell type (Korhonen and Moilanen 2014). Therefore, we measured the effect of rolipram on the levels of phosphorylated p38 and JNK in LPS‐stimulated murine macrophages. Both p38 and JNK were rapidly phosphorylated when the cells were exposed to LPS (Fig. 3). Rolipram significantly reduced the levels of phosphorylated JNK but not the levels of phosphorylated p38 (Fig. 3). This suggests that the effect of rolipram on the expression of mPGES‐1 is specifically mediated by attenuated phosphorylation of MAP kinase JNK rather than p38.

**Figure 4:**
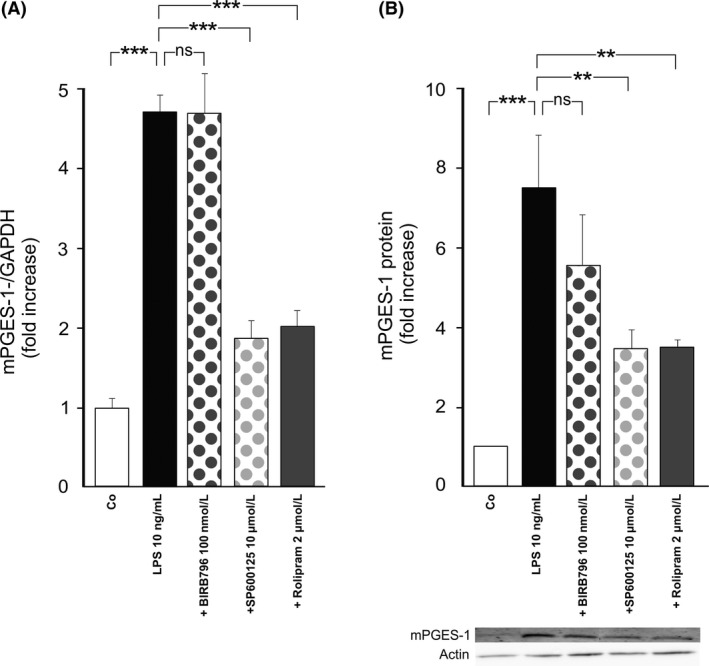
Effects of the selective JNK inhibitor SP600125 and the selective p38 inhibitor BIRB796 on mPGES‐1 expression in J774 murine macrophages. Cells were incubated with bacterial lipopolysaccharide (LPS) and SP600125 or BIRB796 for 24 h. mPGES‐1 mRNA levels were measured by quantitative RT‐PCR and normalized against GAPDH mRNA levels (A). mPGES‐1 protein expression was measured by Western blot where actin was used as a loading control (B). mPGES‐1 expression levels in the unstimulated (control) cells were set as 1 and the other values were related to that. Results are expressed as mean + SEM,* n* = 5–7. One‐way ANOVA with Bonferroni's post‐test was performed and statistical significance is indicated as **P < 0.01, ***P < 0.001 and ns = not significant.

Therefore, we next investigated the effects of a JNK inhibitor compared to those of a p38 inhibitor on J774 macrophages. The JNK inhibitor SP600125 (Bennett et al. 2001) reduced the expression of mPGES‐1 mRNA and protein to a similar extent as rolipram (Fig. 4). In contrast, the p38 inhibitor BIRB796 (Kuma et al. 2005) did not have a significant effect.

**Figure 5:**
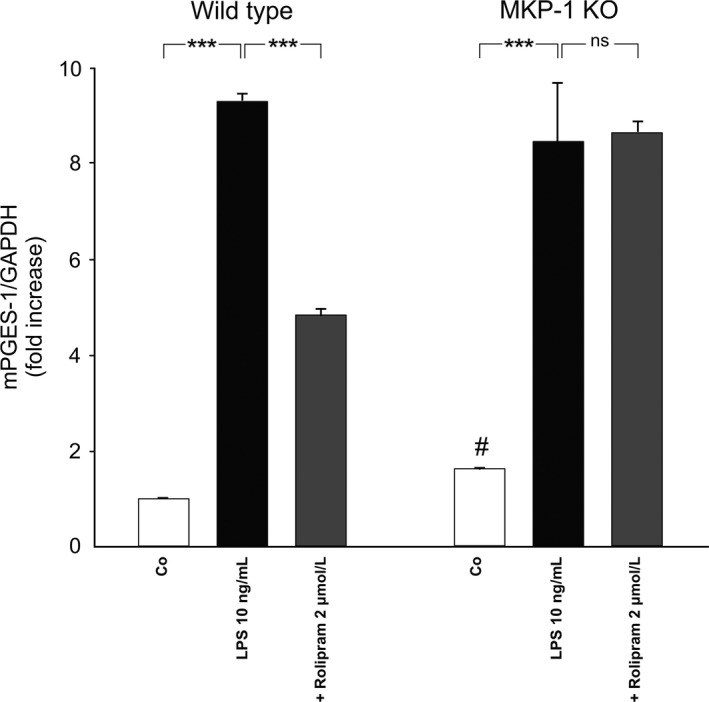
Effects of rolipram on the expression of mPGES‐1 in peritoneal macrophages from wild‐type and MKP‐1‐deficient (knock‐out, KO) mice. Peritoneal macrophages were incubated with bacterial lipopolysaccharide (LPS) or the combination of LPS and rolipram for 24 h. mPGES‐1 mRNA levels were measured by quantitative RT‐PCR and normalized against GAPDH mRNA levels. mPGES‐1 mRNA levels in unstimulated cells from wild‐type mice were set as 1, and the other values were related to that. Results are expressed as mean + SEM,* n* = 4. One‐way ANOVA with Bonferroni's post‐test was performed and statistical significance is indicated as ***P < 0.001, ^#^ *P* = 0.0286 versus unstimulated cells from wild‐type mice and ns = not significant.

Finally, we wanted to investigate the causal role of MKP‐1 as a mediator of the observed drug effect by comparing the effect of rolipram on the expression of mPGES‐1 in peritoneal macrophages from MKP‐1‐deficient and corresponding wild‐type mice. Rolipram significantly decreased the expression of mPGES‐1 in peritoneal macrophages from wild‐type mice, but not in those from MKP‐1‐deficient mice (Fig. 5), whereas mPGES‐1 expression was higher in unstimulated MKP‐1‐deficient macrophages than in wild‐type cells supporting the known role of MKP‐1.

In conclusion, this data suggests that the PDE4 inhibitor rolipram decreases the expression of mPGES‐1 by a mechanism related to increased expression of MKP‐1 and decreased phosphorylation of the MAP kinase JNK in classically activated macrophages.

## Discussion

In this study, we found that rolipram enhanced the expression of MKP‐1 with a concomitant decrease in the expression of mPGES‐1. Furthermore, the inhibitory effect on mPGES‐1 expression seen in peritoneal macrophages from wild‐type mice was abolished in cells from MKP‐1‐deficient mice strongly suggesting that the anti‐inflammatory effect of rolipram on mPGES‐1 expression is indeed dependent of MKP‐1. The results also indicate that excessive mPGES‐1 expression can be downregulated by compounds which enhance MKP‐1 in classically activated macrophages.

Rolipram is a prototypic selective inhibitor of PDE4 with a promising preclinical profile, but the compound was discarded from clinical development due to unfortunate adverse effects (Zhu et al. 2001). Later, rolipram has been widely used as a pharmacological tool in research focusing on effects mediated via suppressed PDE4 activity and enhanced intracellular cAMP levels. Recently, rolipram was found to inhibit the expression of the proinflammatory cytokine TNF‐*α* with a concomitant increase in the expression of the anti‐inflammatory phosphatase MKP‐1 in murine macrophages and to attenuate carrageenan‐induced inflammatory paw edema in wild‐type mice (Korhonen et al. 2013). Both of these effects were abolished in MKP‐1 knock‐out mice proposing a role for MKP‐1 as a mechanism mediating anti‐inflammatory effects of PDE4 inhibitors. This could be explained by the fact that the MKP‐1 promoter contains two cAMP‐responsive elements (CREs) (Kwak et al. 1994) which bind the transcription factor CREB, and the expression of MKP‐1 has been shown to be enhanced by activated cAMP‐PKA‐CREB signaling. In support of that, PDE4 inhibitors, including rolipram, have recently been presented to enhance the anti‐inflammatory effects of *β*_2_‐agonists with a concomitant increase in MKP‐1 expression in airway smooth muscle cells and murine macrophages (Patel et al. 2015; Keränen et al. 2016).

Increased intracellular cAMP is believed to mediate the therapeutic effects of PDE4 inhibitors because they reduce the enzymatic degradation of cAMP. Therefore, it is interesting and seemingly contradictory to our results that elevated cAMP levels triggered by activation of EP2 receptors have previously been reported to increase mPGES‐1 expression in RAW 264.7 macrophages (Diaz‐Munoz et al. 2012). That study proposed a positive feedback process for PGE_2_ production, but PDE4 inhibitors or MKP‐1 were not investigated. In this study, we found that rolipram increased MKP‐1 expression and reduced mPGES‐1 expression both in J774 macrophages and in primary mouse peritoneal macrophages. Based on these studies it is possible that the effects of cAMP on mPGES‐1 expression differ between cell lines or types. On the other hand, the kinetics, intensity and intracellular compartmentalization of increased intracellular cAMP may be different following activation of G‐protein‐coupled receptors and following inhibition of cAMP degrading PDEs. Another possibility is that increased intracellular cAMP has two distinct and opposite effects on mPGES‐1 expression: directly through activation of mPGES‐1 promoter and indirectly through increased MKP‐1 expression. Yet, our results cannot entirely exclude the possibility that the effects of rolipram on MKP‐1 and mPGES‐1 could be cAMP‐independent.

The anti‐inflammatory role of MKP‐1 is suggested to be based on dephosphorylation of MAP kinases p38 and/or JNK depending on the cell type (Franklin and Kraft 1997; Franklin et al. 1998; Korhonen and Moilanen 2014). In addition, mPGES‐1 expression has been reported to be regulated by p38 and/or JNK kinases depending on the cell type, stimulating agent, and signaling cascade concerned. On this account, we wanted to elucidate if the MKP‐1‐dependent effect of rolipram would limit the expression of mPGES‐1 by dephosphorylating p38 or JNK in macrophages. Based on our results, rolipram reduced the levels of phosphorylated JNK but not p38 in association with the enhanced MKP‐1 and suppressed mPGES‐1 expression. This suggests that the downregulation of mPGES‐1 expression by rolipram is mediated via reduced phosphorylation of the JNK kinase. Accordingly, JNK inhibitor was found to suppress the expression of mPGES‐1 in J774 macrophages to an extent comparable with that of rolipram, whereas the specific inhibitor of p38 did not have any significant effect. Apart from our study, the dephosphorylation of JNK as a mechanism behind decreased expression of mPGES‐1 has previously been presented in rat neonatal cardiomyocytes (Degousee et al. 2006), mouse peritoneal macrophages (Cardeno et al. 2014) and recently also in murine microglial cells (He et al. 2016), which findings support our current results.

mPGES‐1 expression is enhanced by a variety of inflammatory factors including IL‐1*β*, TNF‐*α,* and LPS, the latter of which was also used in this study. Early growth response protein 1 (EGR‐1) and nuclear factor kappa B (NF‐*κ*B) are regarded as key transcription factors for induction of mPGES‐1 in inflammation but also other factors have been identified (Koeberle and Werz 2015). One of those is AP‐1 (Moon et al. 2005; Jungel et al. 2007) which is activated by JNK and could explain the JNK‐mediated enhancement of mPGES‐1 found in this study. Another explanation could base on the finding in cardiomyocytes (Degousee et al. 2006) that JNK stabilizes mPGES‐1 mRNA leading to enhanced mPGES‐1 protein levels. Further studies are, however, needed to understand the detailed molecular mechanisms linking JNK activity to enhanced mPGES‐1 expression.

**Figure 6:**
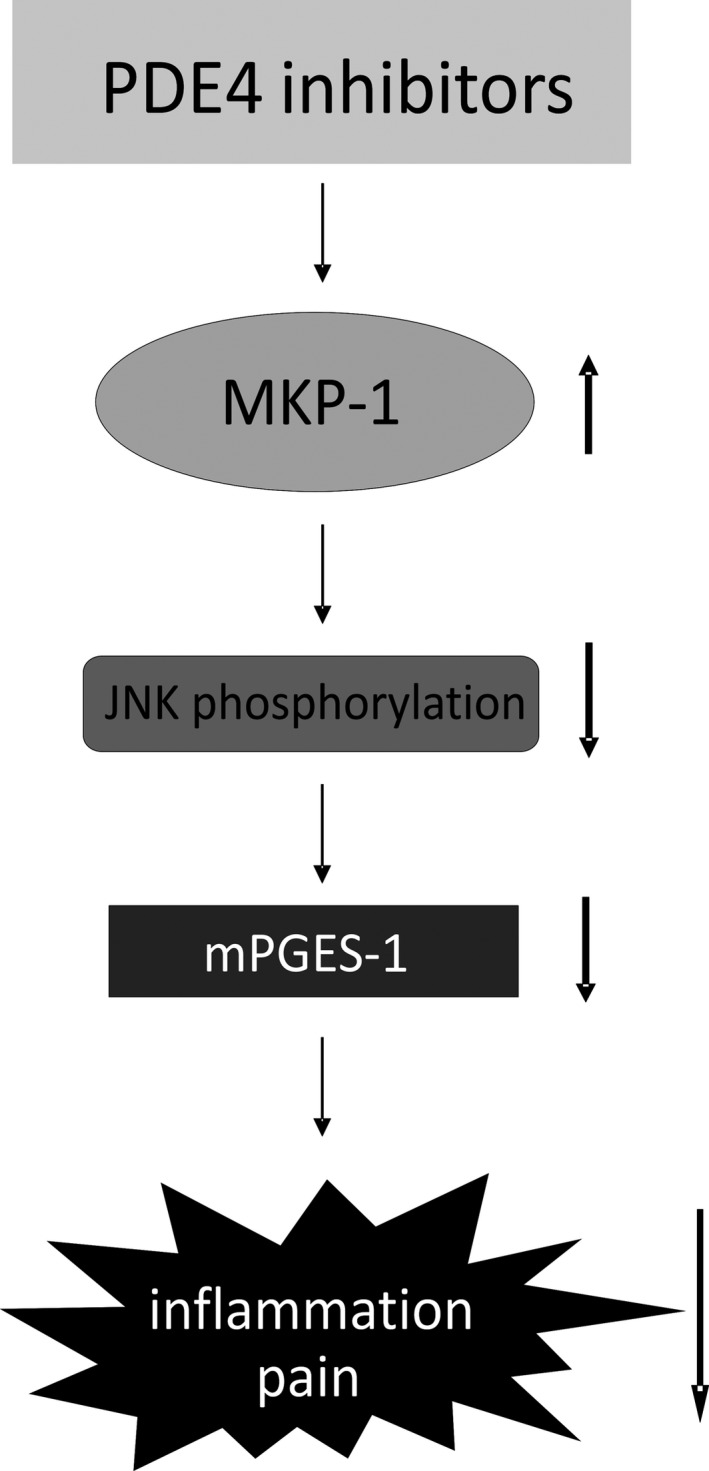
Proposed mechanism of the downregulation of mPGES‐1 by the PDE4 inhibitor rolipram. The PDE4 inhibitor rolipram was discovered to inhibit the expression of mPGES‐1, which is an enzyme closely associated with inflammation and inflammatory pain. The results suggest that the inhibitory effect is mediated via increased expression of MKP‐1 and decreased phosphorylation of MAP kinase JNK. PDE4 =  Phosphodiesterase 4; MKP‐1 =  MAP kinase phosphatase 1; JNK = Jun N‐terminal kinase; mPGES‐1 =  Microsomal prostaglandin E synthase‐1.

In summary, our results show that the PDE4 inhibitor rolipram inhibits the expression of mPGES‐1 in classically activated macrophages. That is a novel anti‐inflammatory activity associated with PDE4 inhibitors, which likely contributes to their clinical efficacy. The mechanism in question was found to be mediated via increased expression of MKP‐1 and decreased phosphorylation (i.e., reduced activity) of the MAP kinase JNK (Fig. 6). These results also underline the significance of MKP‐1 and JNK as targets for development of new anti‐inflammatory treatments targeting conditions complicated with excessive expression of mPGES‐1.

## Disclosure

None declared.
